# Supplementary material for: Recently-adopted foraging strategies constrain early chick development in a coastal breeding gull
Source: PeerJ. 2019 Jul 10;7:e7250. doi: 10.7717/peerj.7250 (PMC6626513; doi:10.7717/peerj.7250)

**Appendix**

Figure A1: composition of chick regurgitates found at Zeebrugge (period 2006-2018)


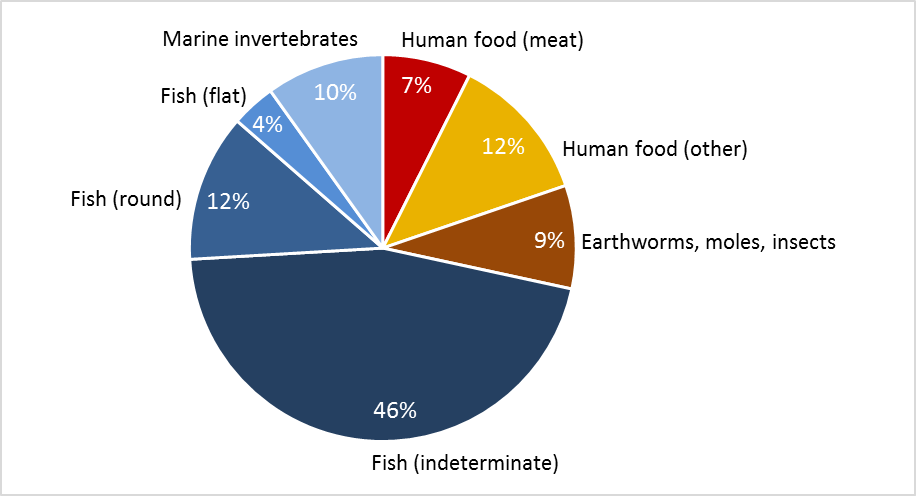


Table A1: Nutrient composition of items found in chick regurgitates from Zeebrugge in the period 2006-2018. Source: Fooddata, Food department, Technical University of Denmark (frida.fooddata.dk).

| Food type | Energy (Kj/100g) | Moisture (g/100g) | Protein (g/100g) | Lipids (g/100g) | Ash (g/100g) | Calcium (g/100g) | Phosphorus (g/100g) | Occurrence |
| --- | --- | --- | --- | --- | --- | --- | --- | --- |
| **Marine Habitat** | | | | | | | | |
| Sprat | 564 | 78 | 10.3 | 10.5 | 1.2 | 0.28 | 0.22 | 4 |
| Sole | 360 | 78.4 | 18.7 | 1.1 | 1.7 | 0.029 | 0.271 | 9 |
| Whiting | 332 | 80 | 18.2 | 0.6 | 1.2 | 0.05 | 0.183 | 45 |
| Shrimp | 336 | 78.7 | 16.5 | 1.2 | 2.9 | 0.078 | 0.119 | 12 |
| Herring | 784 | 68.6 | 16.5 | 13.6 | 1.2 | 0.0338 | 0.2 | 8 |
| Crab | 473 | 72.4 | 22.9 | 1.8 | 1.9 | 0.12 | 0.22 | 4 |
| Mussel | 348 | 80.6 | 11.9 | 2.2 | 1.6 | 0.03 | 0.145 | 8 |
| Scampi | 464 | 70 | 24 | 1.1 | 4 | 0.115 | 0.265 | 2 |
| Cod | 421 | 57.3 | 24.3 | 0.2 | 18.2 | 0.07 | 0.3 | 2 |
| mackerel | 780 | 68.1 | 17.8 | 12.9 | 1.2 | 0.0146 | 0.196 | 3 |
| Lobster | 393 | 76 | 19.6 | 1.3 | 2.4 | 0.048 | 0.144 | 1 |
| Plaice | 369 | 79.4 | 17.5 | 1.9 | 1.1 | 0.0287 | 0.172 | 1 |
| Squid | 385 | 78.55 | 15.58 | 1.38 | <0.01 | 0.032 | 0.221 | 4 |
| **Terrestrial Habitat** | | | | | | | | |
| Chicken (full) | 888 | 65.5 | 17.1 | 15.9 | 0.9 | 0.01 | 0.166 | 5 |
| Chicken butchery | 473 | 76.2 | 18.2 | 4.2 | 0.9 | 0.011 | 0.148 | 2 |
| Bread | 1146 | 33.7 | 8.8 | 3.9 | 1.5 | 0.039 | 0.114 | 19 |
| Potato raw | 326 | 79.5 | 2 | 0.3 | 0.9 | 0.0068 | 0.0553 | 1 |
| Fries | 1299 | 37.1 | 3.7 | 14.8 | 2.2 | 0.0132 | 0.12 | 12 |
| Pork butchery | 432 | 78.6 | 16.1 | 4.3 | 1 | 0.0053 | 0.204 | 3 |
| Pork sausage | 889 | 65 | 12.3 | 16.8 | 2.4 | 0.0407 | 0.245 | 1 |
| sausage mixed | 1108 | 58.3 | 12.5 | 22.6 | 3 | 0.0246 | 0.223 | 1 |
| Rice | 1518 | 10.9 | 7.8 | 1.2 | 0.7 | 0.13 | 0.171 | 2 |
| Spinach | 102 | 91.8 | 2.6 | 0.6 | 1.9 | 0.129 | 0.0413 | 1 |
| Chips | 2221 | 1.3 | 5.4 | 31.9 | 3.7 | 0.024 | 0.148 | 3 |
| Pasta | 1532 | 9.5 | 12.3 | 1.8 | 0.8 | 0.02 | 0.14 | 2 |
| Pastry | 1884 | 12 | 5.8 | 20.9 | 0.9 | 0.033 | 0.097 | 1 |
| Potato cooked | 360 | 77.5 | 1.7 | 0.1 | 0.7 | 0.008 | 0.04 | 1 |
| Bacon | 1374 | 51.9 | 14.8 | 30.3 | 3 | 0.006 | 0.167 | 1 |
| Egg | 608 | 74.2 | 13.7 | 9.5 | 1.2 | 0.0413 | 0.181 | 1 |
| Ham | 467 | 74.7 | 15.7 | 5.4 | 4.2 | 0.006 | 0.2 | 2 |
| TOTAL | | | | | | | | 159 |

Figure A2: *PCA biplot of items found in chick regurgitates from Zeebrugge in the period 2006-2018, based on their nutrient composition.*


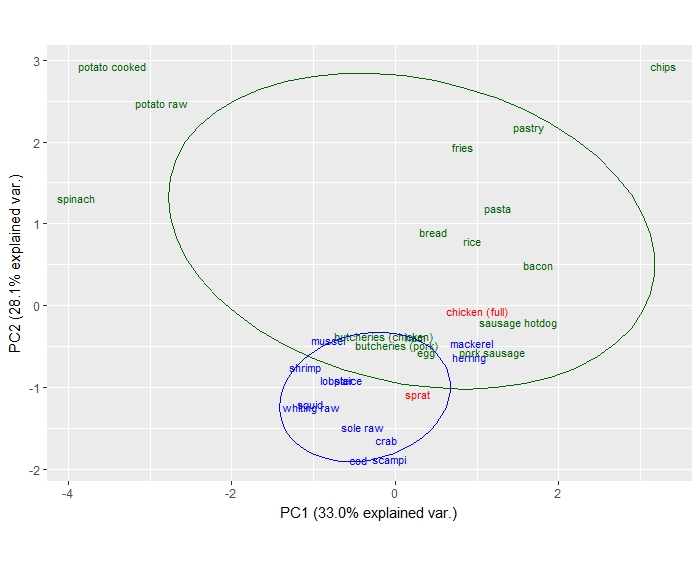


Table A2: TEFs (SD) calculated for fish and chicken on L. fuscus P1 feathers.

| Source | TEF Δ13C | TEF Δ15N |
| --- | --- | --- |
| Fish | 3.0 (.4) | 5.2 (.6) |
| Chicken | 2.0 (.4) | 3.5 (.5) |

Figure A3: Innermost right primary feather length (mm) of chicks raised in the aviary (grey lines), with average values (red line), indicating the averages for 10, 20 and 30 days after hatching.


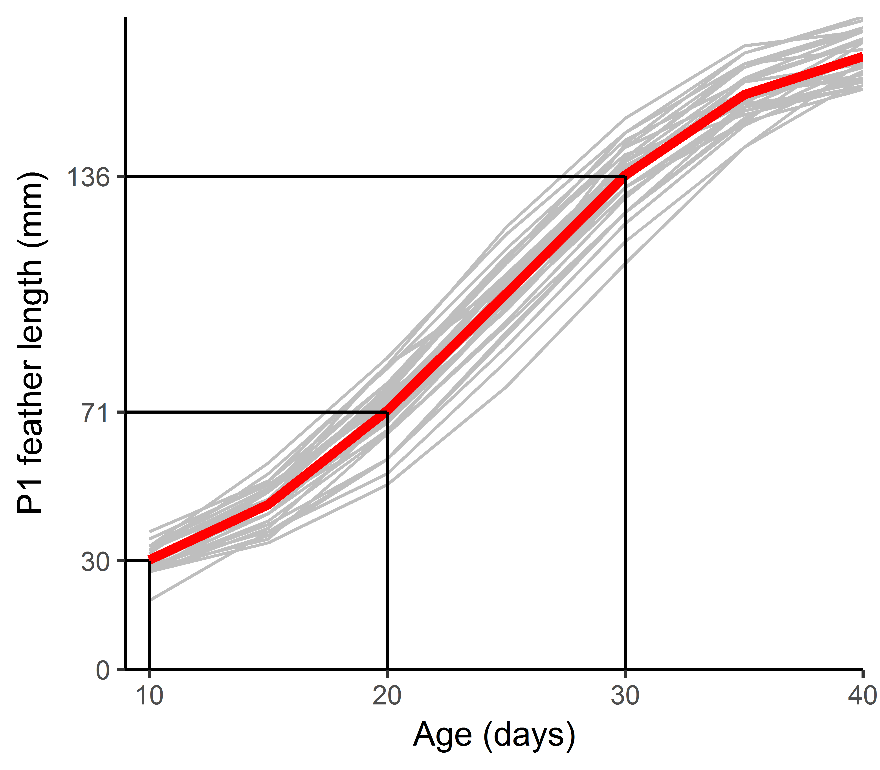

Supplement: Appendix — Fig.5: composition of chick regurgitates found at Zeebrugge (period 2006–2018; Table 4: Nutrient composition of items found in chick regurgitates from Zeebrugge in the period 2006-2018. Source: Fooddata, Food department, Technical University of Denmark (frida.fooddata.dk); Fig. 6: PCA biplot of items found in chick regurgitates from Zeebrugge in the period 2006–2018, based on their nutrient composition. [file peerj-07-7250-s001.docx]
